# A Novel Druggable Dual-Specificity tYrosine-Regulated Kinase3/Calmodulin Kinase-like Vesicle-Associated Signaling Module with Therapeutic Implications in Neuroblastoma

**DOI:** 10.3390/biomedicines12010197

**Published:** 2024-01-16

**Authors:** Esteban J. Rozen, Kim Wigglesworth, Jason M. Shohet

**Affiliations:** 1 Crnic Institute Boulder Branch, BioFrontiers Institute, University of Colorado Boulder, 3415 Colorado Avenue, Boulder, CO 80303, USA; 2 Linda Crnic Institute for Down Syndrome, University of Colorado Anschutz Medical Campus, 12700 East 19th Avenue, Aurora, CO 80045, USA; 3 Department of Pediatrics, University of Massachusetts Medical School, 55 Lake Avenue North, Worcester, MA 01566, USA; kim.wigglesworth@umassmed.edu (K.W.);

**Keywords:** DYRK3, CAMKV, neuroblastoma, cell proliferation, mitotic spindle, biomolecular condensates

## Abstract

High-risk neuroblastoma is a very aggressive pediatric cancer, accounting for ~15% of childhood cancer mortality. Therefore, novel therapeutic strategies for the treatment of neuroblastoma are urgently sought. Here, we focused on the potential implications of the Dual-specificity tYrosine-Regulated Kinase (DYRK) family and downstream signaling pathways. We used bioinformatic analysis of public datasets from neuroblastoma cohorts and cell lines to search correlations between patient survival and expression of *DYRK* kinases. Additionally, we performed biochemical, molecular, and cellular approaches to validate and characterize our observations, as well as an in vivo orthotopic murine model of neuroblastoma. We identified the DYRK3 kinase as a critical mediator of neuroblastoma cell proliferation and in vivo tumor growth. DYRK3 has recently emerged as a key regulator of several biomolecular condensates and has been linked to the hypoxic response of neuroblastoma cells. Our data suggest a role for DYRK3 as a regulator of the neuroblastoma-specific protein CAMKV, which is also required for neuroblastoma cell proliferation. CAMKV is a very understudied member of the Ca^2+^/calmodulin-dependent protein kinase family, originally described as a pseudokinase. We show that CAMKV is phosphorylated by DYRK3, and that inhibition of DYRK3 kinase activity induces CAMKV aggregation, probably mediated by its highly disordered C-terminal half. Importantly, we provide evidence that the DYRK3/CAMKV signaling module could play an important role for the function of the mitotic spindle during cell division. Our data strongly support the idea that inhibition of DYRK3 and/or CAMKV in neuroblastoma cells could constitute an innovative and highly specific intervention to fight against this dreadful cancer.

## 1. Introduction

**Figure 1:**
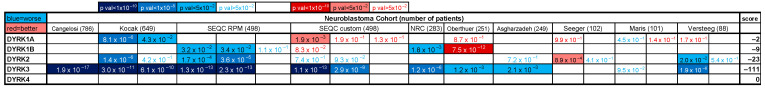
Bonferroni adjusted *p*-values for the correlations between *DYRK* family member expression and NB patient survival probability (pseudo-heatmap). Values highlighted in blue denote significant negative correlations (higher expression = reduced survival probability ‘worse’), while values highlighted in red denote positive correlations (higher expression = increased survival probability ‘better’). Blue/red fonts denote non-significant values (bonf. *p*-value > 5.00 × 10^−2^). Scores are arbitrary units based on the number of datasets showing one or more significant correlations for a given gene, and the value of such significance as previously described [2]. We arbitrarily define that a gene must be significantly correlated to either a better or a worse outcome in at least five independent cohorts, and obtain a minimum of 50 points (either positive or negative, respectively), to be considered a significant hit.

Neuroblastoma (NB) is the most common extracranial solid tumor of childhood, causing about 15% of all pediatric cancer deaths [1]. Despite very aggressive multimodal interventions, only ~50% of high-risk NB patients survive, exhibiting serious long-term sequelae from therapy, warranting innovative and improved interventions. In search of potential druggable targets in NB pathogenesis, we performed a systematic analysis of the correlation between expression of the Dual-specificity tYrosine phosphorylation-Regulated Kinase (DYRK) family members and neuroblastoma (NB) patient survival probability. We annotated and ranked the expression of all five *DYRK* kinases (*DYRK-1A, 1B, 2, 3,* and *4*) across 10 publicly available NB patient datasets from the ‘R2: Genomics Analysis and Visualization Platform’ (as previously described [2]) and observed a significant and robust correlation between *DYRK3* expression and a worse patient survival in 8 of these 10 cohorts, but not for any other family members (Figure 1). This observation was also true for other neurological tumors (particularly Glioma, Supplementary Figure S1).

DYRK3 is a largely understudied kinase. Its expression has been associated with a higher aggressiveness of Glioblastoma cells and tumors [3], although another report suggested it might negatively regulate hepatocellular carcinoma progression [4]. It is noteworthy that Ivanova et al. [5] reported a role for DYRK3 as a negative regulator of the hypoxic response and differentiation in NB cells, hence contributing to their aggressive behavior. DYRK3 remains largely understudied, although recent reports from Lucas Pelkmans’ group and others have implicated this kinase as a critical regulator of several biomolecular condensates, such as Stress Granules [6,7], centrosomes and the mitotic spindle [8], endoplasmic reticulum exit sites [9], or Mediator complex condensates [10]. Biomolecular condensates—also referred to as membranelles organelles—are sub-cellular compartments that form through liquid–liquid phase separation of RNA and/or proteins that contain intrinsically disordered regions (IDRs) [11,12]. Such disordered regions can undergo numerous post-translational modifications, particularly phosphorylation, which regulate their partitioning into these condensates [13,14,15]. We show here that CAMKV is a direct substrate of DYRK3. CAMKV is a protein highly enriched in neuroblastoma cancer cell lines and in healthy neural tissues. Importantly, *CAMKV* levels were previously correlated to a worse NB patient survival and its expression is highly associated to that of *MYCN* or *MYC* in NB cell lines and primary tumor samples [16].

## 2. Materials and Methods

### 2.1. Cell Lines and Culture

The neuroblastoma cell line SJNB-JF-G12 (JF) was originally established in 1979 from a patient with disseminated neuroblastoma and was a kind gift from Dr. Malcom Brenner. The NGP cell line was obtained from DSMZ; SH-SY5Y cells were obtained from ATCC. All cell lines were cultivated at 37 °C with 5% CO_2_. Cell lines were cultured according to vendors’ recommendations and passaged no more than 8 times. JF cells were grown in RPMI 1640 + 10% h.i. FBS + 4 mM L-Glutamine + pen/strept. NGP cells were grown in DMEM (4.5 g/L glucose) + 10% h.i. FBS + 4 mM L-Glutamine + pen/strept. SH-SY5Y cells were grown in DMEM/F12 (1:1) + 10% h.i. FBS + 4 mM L-Glutamine + penicillin/streptomycin. All cell lines were tested for Mycoplasma every 2 months using a e-MycoTM Mycoplasma PCR Detection Kit (Bulldog Bio #25235), according to the manufacturer’s instructions. All cell lines were authenticated by STR analysis (ATCC).

### 2.2. Reagents

Harmine and GSK-626616 were obtained from MedChemExpress (Monmouth Junction, NJ, USA) (HY-N0737A and HY-105309, respectively) and resuspended to a 10 mM stock in DMSO for in vitro/cell culture-based assays. DAPI was from Millipore Sigma (Burlington, MA, USA) (#268298). Primary antibodies from Cell Signaling Technology were: mCherry (#43590), β-actin (#4970), vinculin (#13901), Phospho-PRAS40 (Thr246) (#2997), and HA-tag (#3724). Other primary antibodies were: anti-GAPDH from Millipore Sigma (MAB374), anti-DYRK3 from Aviva Systems Biology (San Diego, CA, USA) (ARP30648_P050), anti-CAMKV from Sino Biological (Wayne, PA, USA) (12243-T26). Mouse anti-rabbit (211-035-109) and goat anti-mouse (115-035-146) HRP-conjugated secondary antibodies were from Jackson ImmunoResearch Laboratories Inc. (West Grove, PA, USA). Purified human recombinant DYRK3 was obtained from Sino Biological (#10726-H20B). Dynabeads Protein A-magnetic beads for immunoprecipitation were obtained from Invitrogen (Waltham, MA, USA) (#10001D). Lipofectamine 2000 transfection reagent was from Invitrogen (#11668027).

### 2.3. Western Blot

Western blot analysis was conducted using standard methods as previously described [17]. Briefly, cells grown to a 60–80% confluency were lysed in radioimmunoprecipitation assay (RIPA) lysis buffer (Prometheus Protein Biology Products #18-416) supplemented with Protease and Phosphatase Inhibitor Cocktails (Pr/Ph-ICs; Pierce, Thermo Scientific A32955 and A32957). Lysates were sonicated on ice, centrifuged at 15,000× *g* at 4 °C for 20 minutes, and the soluble protein fraction was collected. Protein extracts were quantified using a Pierce BCA Protein Assay Kit (Thermo Scientific (Waltham, MA, USA) #23227). A total of 30–50 μg of proteins were separated via SDS-PAGE using Novex™ WedgeWell™ 4–20%, Tris-Glycine Mini Protein Gels (Invitrogen, Thermo Scientific XP04202BOX) and blotted onto a PVDF membrane using an iBlot transfer system and transfer stacks (Invitrogen, Thermo Scientific IB401001). Proteins were detected using SuperSignal™ West Pico PLUS Chemiluminescent Substrate (Thermo Scientific 34580). A ChemiDoc MP Imaging System (Bio-Rad, Tokyo, Japan) was used for chemiluminescent detection and analysis. 

### 2.4. Immunoprecipitation and In Vitro Kinase Assay

For soluble cell lysates, cells were washed twice in PBS and lysed in IP buffer (50 mM Tris-HCl [pH 7.4], 150 mM NaCl, 2 mM EDTA, 1% NP-40, 10% glycerol, Pr/Ph-ICs). Lysates were clarified by centrifugation and protein quantification with the BCA assay kit (Pierce). One mg of total protein/sample was incubated for 6 h at 4 °C with protein A/G-magnetic beads (Dynabeads, Invitrogen, ThermoFisher Scientific (Vilnius, Lithuania)) prebound with 5 μg of anti-mCherry antibody, and then beads were washed with IP buffer. Finally, both lysates (input) and the immuno-precipitates were resuspended in 5× LB and analyzed by Western blotting. For in vitro kinase (IVK) from immunocomplexes, cell lysates were prepared in IP lysis buffer (50 mM HEPES [pH 7.4], 75 mM NaCl, 1 mM EDTA, 1% NP-40, Pr/Ph-ICs). Cell lysates were incubated overnight at 4 °C with anti-mCherry bound to protein A-magnetic beads (Dynabeads, Invitrogen). Immunocomplexes were washed 4 times with IP and twice time with kinase buffer (25 mM Hepes pH 7.4, 5 mM MgCl_2_, 5 mM MnCl_2_, 0.5 mM DTT). Anti-mCherry immuno-complexes were split into 3 aliquots: 5% for Western blotting and 2 aliquots of 45% for IVK assay, with and without human recombinant DYRK3. Immunocomplexes were incubated for 20 min at 30 °C in 30 μL of kinase buffer with a final concentration of 50 μM ATP and [γ-^32^P] ATP (1 × 10^−2^ μCi/pmol, PerkinElmer (Shelton, CT, USA)). Reactions were stopped by adding 5× LB, and samples were resolved by SDS-PAGE and then stained with Coomassie blue. Incorporation of ^32^P was detected by autoradiography of dried gels.

### 2.5. Orthotopic Xenograft Renal Capsule Injection

One million Tet-shDYRK3 JF cells suspended in 0.1 mL of PBS were surgically implanted in the left renal capsule of NSG immunodeficient mice (The Jackson Laboratory (Bar Harbor, ME, USA) #005557), as previously described [18]. After 7 days, mice were randomly divided into ‘control’ (untreated) group vs. ‘doxy’ group, receiving 1 mg/mL doxycycline in the drinking water for 3 additional weeks. The body weight of mice was monitored weekly. At the end of the treatment, all mice were euthanized. Tumors and the right kidneys (control) were dissected and weighed. All procedures were approved by the Institutional Animal Care and Utilization Committee (IACUC) at UMass Chan Medical School and according to our IACUC approved protocol (A3306-01). 

### 2.6. RT-qPCR

Total RNA was extracted from cells using the Quick-RNA MiniPrep Kit (Zymo Research (Irvine, CA, USA) #11-327) following the manufacturer’s protocol. cDNA was synthesized from 0.5 ug/sample of total RNA with the ABScript II RT Mix (ABclonal (Woburn, MA, USA) RM21452) according to the manufacturer’s instruction. The cDNA was amplified in 96-well reaction plates with a Universal SYBR Green Fast qPCR Mix (ABclonal RM21203) on a QuantStudio 3 real-time PCR system (Applied Biosystems (Waltham, MA, USA), Thermo Fisher Scientific). The sequences of forward and reverse primers are available in the ‘Supplementary Material’ section. The relative level of target transcripts was calculated from triplicate samples after normalization against human TBP and/or GAPDH transcripts. Dissociation curve analysis was performed after PCR amplification to confirm primers’ specificity. Relative mRNA expression was calculated using the ΔΔCT method.

### 2.7. Cell Proliferation Analysis

Cell proliferation of indicated conditions (untreated vs. shRNA-expressing cells) was measured as the relative whole-well confluency of 96-well culture plates using a Celigo Imaging Cytometer (Nexcelom Bioscience LLC, Lawrence, MA, USA). Briefly, ~1000 cells/well were plated on day-1 and incubated for 24 h to allow the cells to attach and recover. The following day each well was imaged and analyzed for relative confluence (day 0). After imaging, cells were treated with vehicle (DMSO, ‘untreated control’) or the indicated final concentrations of Harmine or Doxycycline for the indicated times. Relative confluence was subsequently analyzed every 24/48 h until untreated control wells reached 70–90% confluency. Each condition was performed in 3–6 replicates per experiment. Medium +/− treatment was changed every 48–72 h. Cell proliferation was plotted as the time-dependent change of the average relative confluency for each condition using GraphPad Prism 8 software.

### 2.8. Immunofluorescence Analysis

Standard immunofluorescence techniques were used as recommended in the Cell Signaling Technology protocols webpage. Briefly, cells were fixed in 4% paraformaldehyde in PBS for 15 min at room temperature or in 100% ice-cold methanol for 15 min at 4 °C (for anti-CAMKV), washed 2× in PBS (5 min/wash), and 2× with 0.2% Triton X-100 in PBS (‘PBS-T’). Cells were blocked in 2% BSA-PBS-T (blocking buffer) for 60 min at 4 °C and incubated with primary antibodies diluted in blocking buffer overnight at 4 °C. Then, 4× washes in PBS and 1 h incubation with secondary antibody Alexa 488 goat anti-rabbit (Invitrogen) followed by 4× additional washes in PBS. Images were obtained in an Echo Revolve fluorescence microscope (BICO (San Diego, CA)) or in a Zeiss LSM700 (Peabody, MA) confocal microscope (UMass Chan Medical School). Images of mCherry-expressing constructs were obtained from in vivo microscopy with the Echo Revolve fluorescence microscope.

### 2.9. Lentivirus Preparation and Infection

HEK-293T cells were maintained at 37 °C in Dulbecco’s modified Eagle medium (DMEM), supplemented with 10% FCS and antibiotics (100 units/mL penicillin and 100 μg/mL streptomycin). Cells were transfected with pVSV-G [19] and pCMV∆R8.91 [20], together with the pLKO.1-puro non-targeting vector (Sigma Mission clone SHC016; ‘shControl’) or the pLKO.1-puro-shRNA vectors to target DYRK3 or CAMKV (Sigma Mission clone numbers available in the ‘Supplementary Material’ section, obtained from the UMass Chan Medical School RNAi core facility) using Lipofectamine^TM^ 2000 reagent (Invitrogen) as recommended by the fabricant, and following the recommendations of The RNAi Consortium (TRC) laboratory protocols with slight modifications. Twelve hours after transfection the medium was replaced by DMEM, supplemented with 30% FCS and antibiotics. Cell supernatants were harvested every 24 h, replaced with fresh medium, and stored at 4 °C until collection of the last harvest (at 72 h). At this point, the consecutive harvests were pooled, filtered through 0.45 μm filters, and split in 3–5 mL aliquots, which were stored at −80 °C. NB cells were infected with shControl or shRNA lentiviral particles by adding a 1:1 mix of medium:viral supernatant for 24–48 h. Puromycin selection (2 μg/mL) was applied for 2–3 days and always compared to non-transduced control cells, which generally died within the first 24 h. Target downregulation was confirmed by Western blot and/or RT-qPCR. For mCherry overexpressing constructs the same lentiviral production strategy was followed, using a lentiviral expression construct instead of the pLKO.1-puro shRNA vectors. pLV-mCherry-CAMKV (WT) under the medium-strength promoter EFS (Human eukaryotic translation elongation factor 1 α1 short form) was custom designed and ordered from VectorBuilder. ‘mCherry-only’ and ‘mCherry-CAMKV-∆IDR’ variants were cloned from the original pLV-mCherry-CAMKV construct by standard molecular biology techniques. For the tetracycline-inducible shDYRK3 system, the Tet-pLKO-puro vector was obtained from Addgene (#21915) and cloning of the shDYRK3-2 hairpin (TRCN0000000647) was performed as recommended in the Tet-pLKO manual (available at www.addgene.org). Cells expressing mCherry were purified by FACS at the UMass Chan FACS core facility. All constructs were confirmed by Sanger sequencing.

### 2.10. Statistical Analyses

All quantitative data points represent the mean of three independent experiments performed in 3 or more replicates with standard deviation (S.D). Statistical analysis was performed using a *t*-test or two-way ANOVA (GraphPad Software, Inc., La Jolla, CA, USA).

## 3. Results

**Figure 2:**
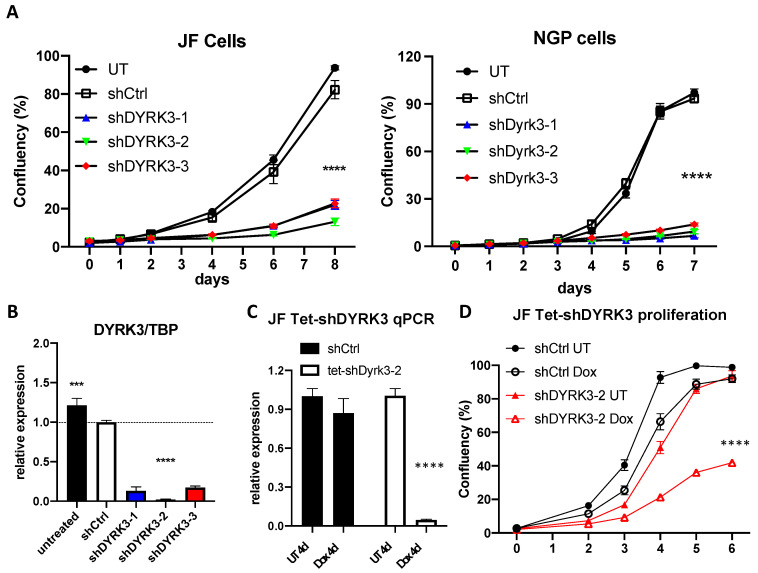
(**A**) NGP and JF neuroblastoma cells were left untreated (UT) or transduced with lentiviral vectors for expression of the indicated shRNAs. After puromycin selection, ~2 × 103 cells were plated on M96 well plates (4 replicates/condition) and allowed to grow for 8 days. Confluency of each well was measured in an Image cytometer (Celigo, Nexcelom). (**B**) JF cells form A were collected in triplicate and processed for RNA extraction and qRT-PCR analysis of *DYRK3* expression (vs. the housekeeping gene *TBP*). (**C**) Tetracycline-inducible shRNA system validation by qRT-PCR for *DYRK3*/*TBP* expression demonstrated efficient and specific *DYRK3* downregulation upon doxycycline treatment (1 μg/mL) of Tet-shDYRK3-expressing cells. (**D**) Tet-shRNA-expressing cells (shCtrl vs. shDYRK3) were plated on M96 well plates (4 replicates/condition) and allowed to grow for 6 days in the absence or presence of doxycycline. Confluency of each well was measured as in A. *** = *p* value > 0.001; **** = *p* value > 0.0001.

To investigate in more detail the relation between DYRK3 levels and neuroblastoma pathophysiology, we knocked down its expression by means of three specific shRNA-expressing lentiviral clones (shDYRK3-1/2/3) vs. a non-targeting control (shCtrl) in two different NB cell lines. In this way, we observed a very striking impairment in NB cell proliferation (Figure 2A). Efficient and specific downregulation of *DYRK3* was confirmed by quantitative Real-Time PCR (RT-qPCR; Figure 2B and Figure S2), demonstrating a critical role for DYRK3 in neuroblastoma cell homeostasis. To further examine DYRK3 implications in NB tumorigenesis, we established a tetracycline-inducible shDYRK3 expression system (Tet-shDYRK3 JF cells). Validation of this paradigm by RT-qPCR and Western blot (WB) confirmed efficient downregulation of DYRK3 upon doxycycline treatment (Figure 2C and Figure S3), again resulting in significantly impaired cell proliferation (Figure 2D). Treatment of JF NB cells with low doses (1 μM) of Harmine, a pan-DYRK inhibitor known to block DYRK3, resulted in a moderate but significant inhibition of cell proliferation (Supplementary Figure S4). At higher doses (10 μM) all cells died within 48 h, suggesting a non-specific cytotoxic effect.

**Figure 3:**
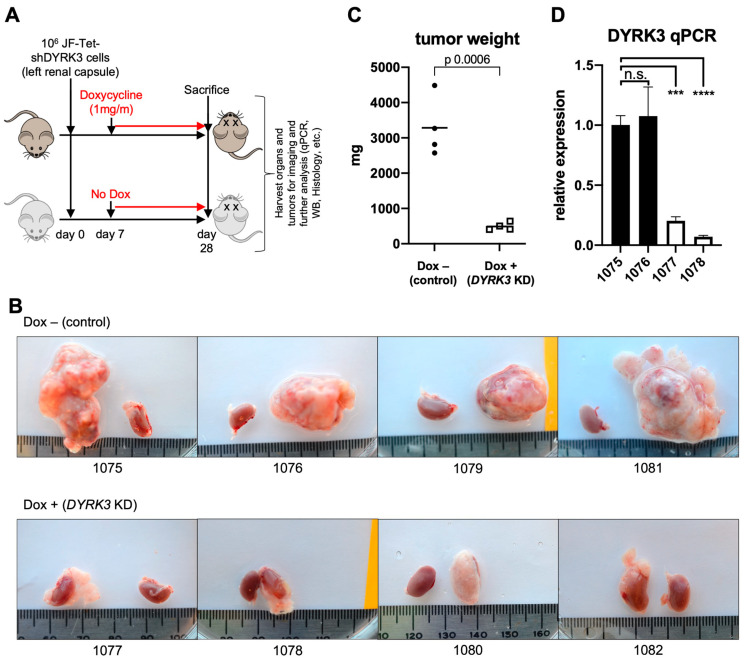
(**A**) Experimental design for assessment for in vivo examination of DYRK3 implications on NB tumor growth. (**B**) Pictures of JF-derived renal tumors and corresponding contralateral kidneys (as control) at experimental endpoint (28 dps), showing the striking defect on tumor growth by tetracycline-inducible DYRK3 downregulation (n = 4 animals/group; see main text). (**C**) Tumor growth inhibition was quantified as tumor weight, showing a very significant reduction in tumor mass by DYRK3 downregulation. (**D**) RNA was isolated from 2 tumors per group and subjected to qRT-PCR to quantify *DYRK3* expression, demonstrating efficient *DYRK3* downregulation by doxycycline treatment. n.s. = not significant; *** = *p* value > 0.001; **** = *p* value > 0.0001.

To assess the direct role of DYRK3 in NB tumorigenesis in vivo, Tet-shDYRK3 JF cells were orthotopically injected into the renal capsule of immuno-compromised NSG^TM^ recipient mice. Seven days post-surgery (7 dps), mice were randomly divided into a ‘control’ (untreated) group vs. ‘doxy’ group, receiving 1 mg/mL doxycycline in the drinking water for three additional weeks (Figure 3A). Importantly, all mice in the control group developed large tumors, while the doxy group tumors were dramatically smaller (Figure 3B,C). As a control, the contralateral healthy kidneys showed no significant weight differences (Supplementary Figure S5). RT-qPCR analysis from tumor RNA samples reflected the efficient downregulation of *DYRK3* by doxycycline treatment (Figure 3D), thus confirming a critical and previously unrecognized role for this kinase in NB cell proliferation and in vivo tumor growth. As mentioned above, Ivanova et al. [5] reported a role for DYRK3 as a negative regulator of the hypoxic response and differentiation in NB cells, hence contributing to their aggressive behavior. Surprisingly, this work was entirely based on DYRK3 endogenous or ectopic expression, but no pharmacological inhibition or downregulation was performed, and it did not examine potential effects on NB cell proliferation. Hence, our robust preliminary results are not in disagreement with such a function in a hypoxic setting.

In search of a mechanistic target, we explored the literature for known DYRK3 substrates that could have a specific role in NB tumorigenesis. Wippich et al. [6] performed an in vitro kinase substrate identification screen using protein microarrays in the presence of wild-type (WT) recombinant DYRK3 vs. a kinase dead mutant (K128M) as a negative control. Among the 26 protein target hits phosphorylated only by WT DYRK3, they found FIP1L1, AKT1S1, and CAMKV as the top three candidates by average Z-score. The authors went on to characterize AKT1S1 (PRAS40) as a novel phosphorylation substrate of DYRK3 in stress granule biology. 

**Figure 4:**
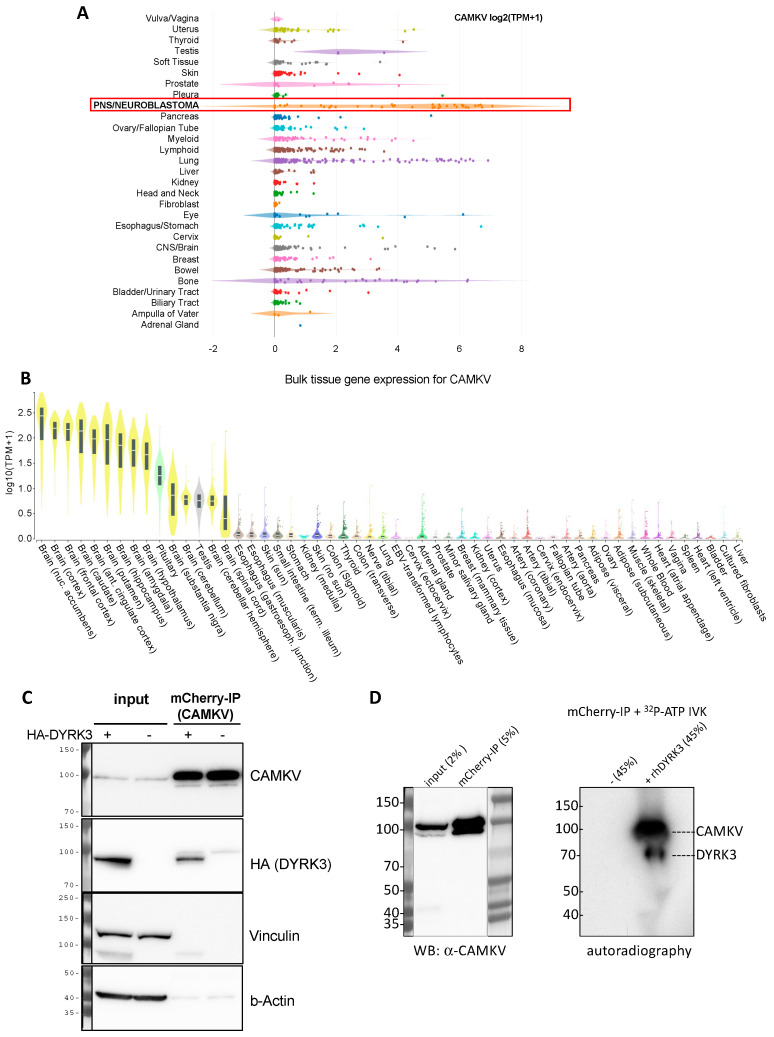
(**A**) Expression of *CAMKV* across different cancer cell lines from the depmap portal (depmap.org) demonstrates a high and specific expression in neuroblastoma cell lines (red frame) as compared to most other cancer cell lines. (**B**) Analysis of *CAMKV* expression across human tissues from the Genotype-Tissue Expression Portal (gtexportal.org) demonstrates that *CAMKV* expression is exclusively enriched in brain tissues. (**C**) Immunoprecipitation studies in NGP NB cells overexpressing mCherry-CAMKV WT +/− HA-DYRK3 demonstrate DYRK3 (HA) co-precipitation with mCherry (CAMKV) immuno-complexes. (**D**) In vitro kinase assay with radioactive [γ-^32^P]-ATP, in the absence or presence of recombinant human DYRK3 confirmed the direct in vitro phosphorylation of CAMKV by DYRK3.

We thus became interested in CAMKV, which was previously correlated to a worse NB patient survival and whose mRNA expression is highly associated to that of *MYCN* or *MYC* in NB cell lines and primary tumor samples [16]. The Calmodulin Kinase-like Vesicle-associated (CAMKV) protein is a member of the Ca^2+^/calmodulin-dependent protein kinase family, highly enriched in brain and endocrine tissues [21]. Due to a lack of structural conservation in key residues, CAMKV is predicted to have impaired kinase activity, although experimental validation of this idea awaits full validation. In agreement with a previous report [16], we observed that *CAMKV* is highly enriched in neuroblastoma cancer cell lines (Figure 4A) and in healthy neural tissues (Figure 4B).

We thus decided to explore in more detail the potential relation between DYRK3 and CAMKV in NB cell homeostasis. To corroborate that CAMKV indeed can interact with DYRK3, we used the NGP NB cell line transduced with a lentiviral vector to stably over-express an mCherry-CAMKV fusion protein. These cells were transiently transfected with an HA-tagged DYRK3 WT (HA-DYRK3) construct or an empty vector as control, and the corresponding cell lysates were subjected to immuno-precipitation (IP) with an mCherry-specific antibody. Western blot analysis of these immuno-precipitates revealed a clear anti-HA signal in the HA-DYRK3-tranfected sample, but not in the control, demonstrating DYRK3 coprecipitation with CAMKV (Figure 4C). Finally, mCherry immuno-precipitates from NGP mCherry-CAMKV cells were subjected to in vitro kinase assays (IVKs) with radioactive [γ-^32^P]-ATP, in the absence or presence of recombinant human DYRK3. This approach confirmed the direct in vitro phosphorylation of CAMKV by DYRK3 (Figure 4D).

**Figure 5:**
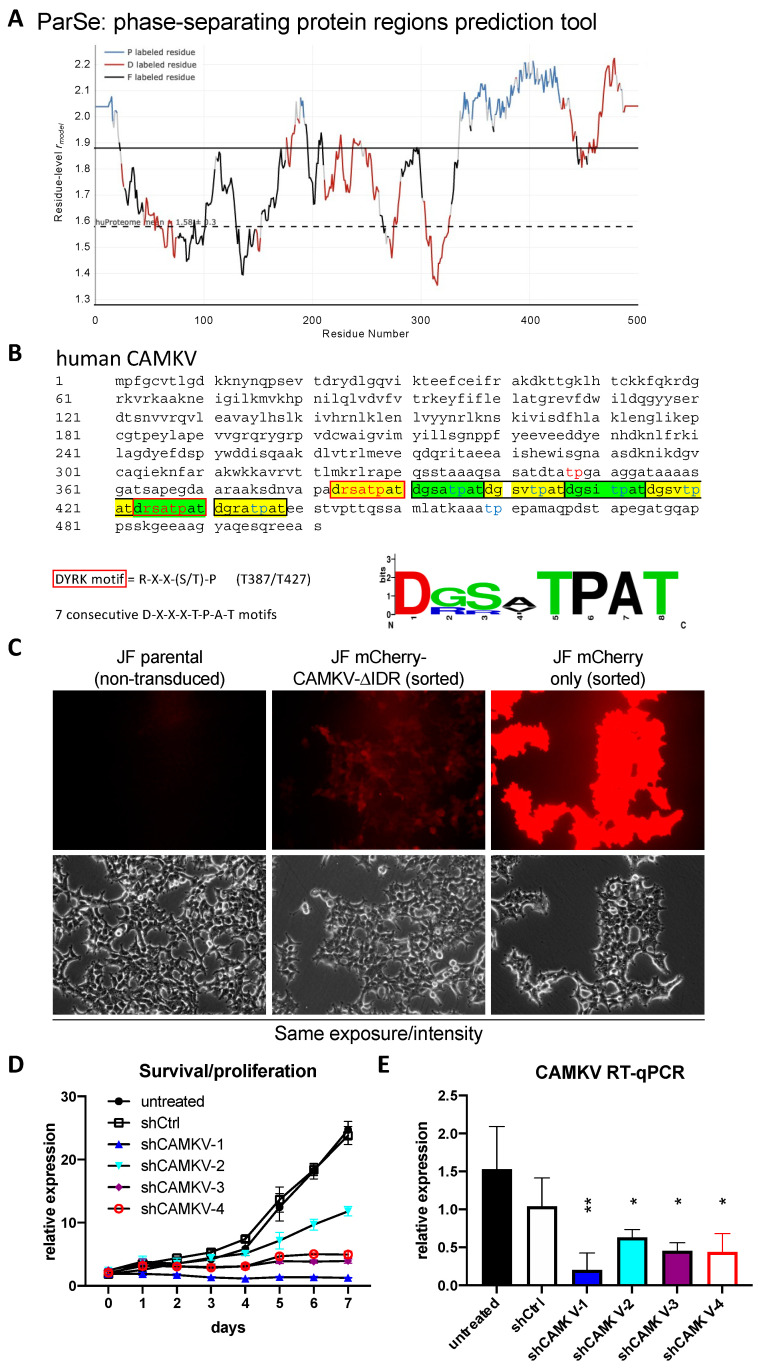
(**A**) CAMKV protein sequence (Accession: NP_076951.2) was analyzed with the phase-separating protein regions online prediction tool, ParSe. P Regions (P) are intrinsically disordered and prone to undergo LLPS. D Regions (D) are intrinsically disordered but do not undergo phase separation. F Regions (F) may or may not be intrinsically disordered but can fold to a stable conformation. (**B**) CAMKV protein sequence (Accession: NP_076951.2) depicting the 7 tandem octapeptide motifs (D-X-X-X-T-P-A-T), including 2 canonical DYRK phosphorylation sites (T387 and T427, red fonts). (**C**) JF neuroblastoma cells were left untreated (parental) or transduced with lentiviral vectors for overexpression of the indicated construct. Cells were then sorted for purity. Cells transduced with mCherry-CAMKV-ΔIDR had a very low mCherry signal, suggestive of a highly unstable product. Scale bar = 40 μm. (**D**) JF cells were left untreated or transduced with indicated shRNA-expressing lentiviral vectors. Cells were selected with puromycin for 3–4 days and then subjected to time-course proliferation assays (4 replicates/condition). Confluency of each well was measured in an Image cytometer (Celigo, Nexcelom), demonstrating a severe impairment in cell proliferation by CAMKV downregulation. (**E**) The same cells used in D were processed in parallel for RNA extraction and subjected to RT-qPCR to confirm *CAMKV* mRNA downregulation. * = *p* value > 0.1; ** = *p* value > 0.01.

Given the known roles for DYRK3 as a critical regulator of the liquid–liquid phase separation (LLPS) behavior of its substrates, we then focused on CAMKV’s protein sequence. Barylko et al. [21] recently reported a predicted intrinsically disordered region of ~200 amino acids in CAMKV’s C-terminal half. We employed bioinformatic tools for the prediction of LLPS formation —ParSe (phase-separating protein regions prediction tool; Figure 5A)— or for prediction of disordered regions —PONDR^®^ (Predictor of Natural Disordered Regions) and PrDOS (Protein DisOrder prediction System; Supplementary Figure S6)—, which further confirmed the presence of a highly disordered C-terminal region likely capable of undergoing phase separation in a phosphorylation-regulated manner. Upon a deeper look, we identified seven tandem repeats of an octapeptide motif (D-X-X-X-T-P-A-T), including two canonical and five highly related DYRK phosphorylation motifs (Figure 5B). In fact, in the original characterization of rat Camkv, Godbout et al. [22] briefly described such region as a potential ‘PEST sequence’, a motif known to act as a signal for degradation in proteins with a short half-life [23,24]. In this context, our overexpression experiments with the lentivirally encoded mCherry-CAMKV WT construct suggested CAMKV as a very stable protein (see below), as demonstrated by the high and constant expression of mCherry-CAMKV across many passages with no loss (but rather increase) of the signal with time. Furthermore, in an attempt to characterize the role of these unique tandem motifs, we generated an overexpression mutant version, ‘mCherry-CAMKV-∆IDR’, lacking the 56 amino acids corresponding to the seven tandem octapeptide repeats. Surprisingly, the mutant variant exhibited a very low to null expression, suggesting that the resulting protein was very unstable (Figure 5C), and confirming that the seven tandem octapeptide repeats are needed for expression or stabilization—and likely function—of CAMKV, and not acting as a canonical PEST sequence. Interestingly, shRNA-mediated downregulation of *CAMKV* by shRNA lentiviral vectors also resulted in a striking impairment of NB cell proliferation (Figure 5D). Of note, the level of *CAMKV* downregulation by different shRNA efficiencies (Figure 5E) was nicely correlated to the degree of proliferation potential of these cells (Figure 4D), suggesting that CAMKV is required for NB cell proliferation or survival in an expression level-dependent fashion.

To examine whether CAMKV might form or localize to membraneless organelles, we analyzed our mCherry-CAMKV overexpressing cells by fluorescence microscopy. CAMKV was originally predicted to lack a kinase activity and associated with neuronal vesicles of the rat cortex [22]. More recent work in mouse models implies a role in activity-dependent bulk endocytosis during the recycling of synaptic vesicles [25], while Liang et al. [26] suggested the co-localization of Camkv with postsynaptic scaffold protein PSD-95 puncta, where it may be required for the activity-dependent maintenance of dendritic spines. Nevertheless, in those immunofluorescent staining images, Camkv was not specifically localized to the dendritic spines, but rather homogeneously distributed all along the neuron. Additionally, Barylko et al. [21] found that murine Camkv can undergo palmitoylation on its N-terminal end, and that this modification was necessary for plasma membrane localization of a Camkv-EGFP (C-terminal) fusion construct. Interestingly, the authors noted that an N-terminal fusion construct (EGFP-Camkv) did not localize to the membrane, but had a homogeneous cytosolic distribution. As for human CAMKV, Sussman et al. [16] also suggested a membrane localization in NB cell lines, although their data were largely inconclusive. 

**Figure 6:**
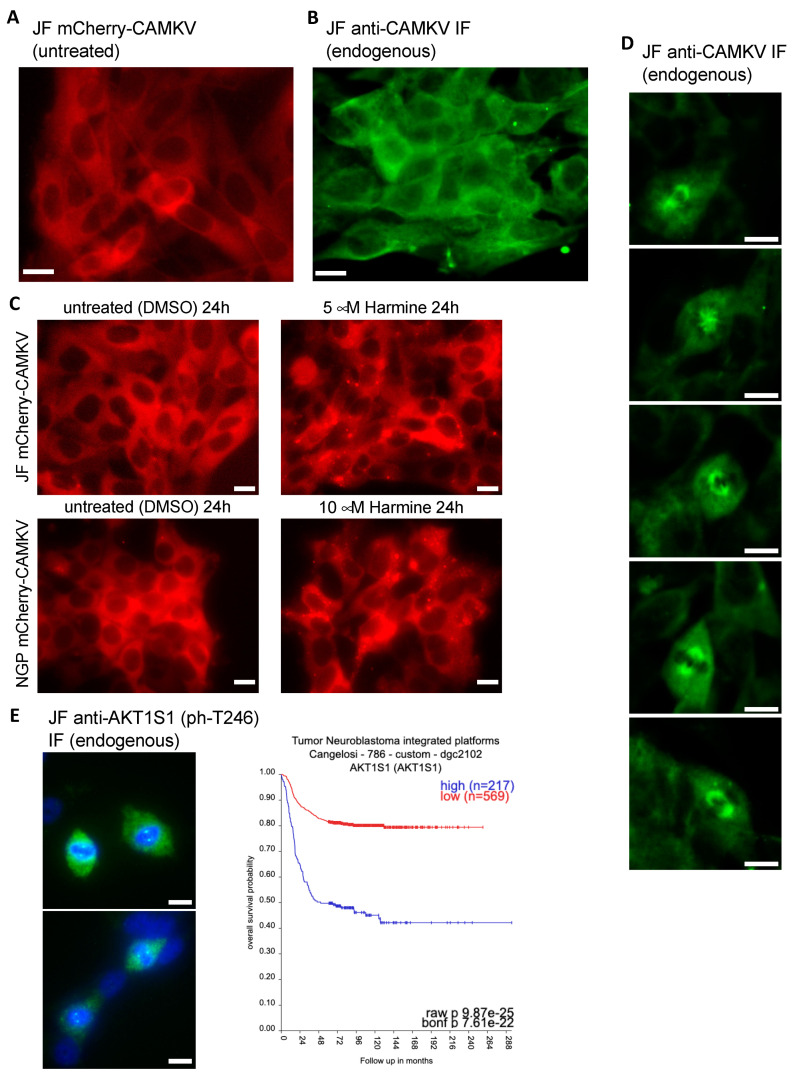
(**A**) Fluorescence image of live JF cells transduced with a lentiviral vector for expression of mCherry-CAMKV (WT), demonstrating a homogeneous cytosolic distribution of the mCherry signal. (**B**) JF cells were fixed and processed for fluorescent immuno-staining of endogenous CAMKV, again showing a widespread cytosolic localization in interphasic cells. (**C**) mCherry-CAMKV JF and NGP cells were left untreated (left panels) or treated with the indicated final concentrations of Harmine for 24. Harmine treatments induced a relocalization of CAMKV from a homogeneous distribution into numerous aggregates (see Supplementary Videos and Figure S7). (**D**) Same cells from (**B**) demonstrating a clear mitotic spindle localization of CAMKV in cells undergoing cell division. (**E**) Left panel: same cells from (**B**,**D**) were processed for fluorescent immuno-staining of endogenous phospho-AKT1S1 (Thr 246), showing a clear mitotic spindle pole localization in cells undergoing cell division. Scale bar = 10 μm. Right panel: Kaplan–Meier plot for the correlation between *AKT1S1* expression and NB patient survival probability in the Cangelosi 786 cohort, as obtained from the ‘R2: Genomics Analysis and Visualization Platform’ (https://hgserver1.amc.nl/cgi-bin/r2/main.cgi (accessed on 2 September 2023)).

In our hands, ectopic expression of mCherry-CAMKV in NB cell lines showed a clear homogeneous cytosolic pattern (Figure 6A), consistent with that reported by Barylko et al. [21] for EGFP-Camkv. More importantly, immuno-fluorescent staining of endogenous CAMKV confirmed a similar cytoplasmic distribution in interphasic NB cells (Figure 6B). Since inhibition of DYRK3 has been shown to affect the organization of several biomolecular condensates [6,7,8,9,10], we treated mCherry-CAMKV JF and NGP cells with Harmine, a pan-DYRK inhibitor known to block DYRK3. Interestingly, this treatment resulted in the relocalization of mCherry-CAMKV into numerous aggregates (Figure 6C and Figure S7) with a very dynamic behavior (Supplementary videos S1 and S2). Treatment of these cells with an unrelated DYRK3 inhibitor, GSK-626616, showed similar effects (Supplementary Figure S8). These results are consistent with previous observations in other biomolecular condensates upon DYRK3 inhibition [6,8,9], and suggest that CAMKV might indeed undergo liquid–liquid phase separation in a DYRK3-regulated fashion. 

It is noteworthy that we failed to observe aggregation of endogenous CAMKV upon Harmine treatment. This might suggest that the mCherry-CAMKV aggregates are a result, at least in part, from its non-physiological over-expression, or that endogenous CAMKV aggregates are sensitive to the harsh methanol fixation used for this staining. Importantly, in our immuno-fluorescence staining analyses of endogenous CAMKV we noticed that virtually every NB cell undergoing cell division displayed a considerably higher anti-CAMKV signal as compared to neighboring interphasic cells. Surprisingly, this signal corresponded to a very clear staining of the mitotic spindle (Figure 6D and Supplementary Figure S9), a transitory structure fundamental for the progression of the cell cycle, and whose organization is governed by liquid–liquid phase separation [27,28,29]. As mentioned above, Rai et al. [8] reported DYRK3 colocalization with Pericentrin in the mitotic spindle poles, further supporting a potential direct role in the regulation of CAMKV function during cell division. Interestingly, we also observed a clear localization in the mitotic spindle poles for endogenous phospho-Thr246 AKT1S1 in dividing JF cells (Figure 6E left panel). As also mentioned above, Wippich et al. [6] characterized the direct phosphorylation of Thr246-AKT1S1 by DYRK3, although a function for this signaling module in the regulation of the mitotic spindle has never been reported. Importantly, we further noticed that *AKT1S1* expression is also highly correlated to a worse NB patient outcome (Figure 6E right panel), as has been suggested for several other cancer types [30]. This observation supports the idea that DYRK3 might act as a central orchestrator of the mitotic spindle organization by recruiting and/or phosphorylating CAMKV and AKT1S1. Therefore, we propose that downregulation or pharmacologic inhibition of DYRK3, CAMKV, and AKT1S1 in NB cells results in an impairment of the mitotic spindle organization and subsequent exit from the cell cycle, a function that will be the focus of future work.

## 4. Discussion

In the present study we provide robust evidence demonstrating a role for the DYRK3 kinase as a critical driver of neuroblastoma cell proliferation and tumor growth. Our results suggest that this activity might be associated to the ability of DYRK3 to interact with and phosphorylate CAMKV, a protein abundantly expressed in high-risk NB and—like DYRK3—strongly correlated to a worse patient survival probability. Unfortunately, specific DYRK3 inhibitory compounds are currently lacking. Our data are consistent with a role for DYRK3 in the regulation of CAMKV partitioning into liquid–liquid phase separated biomolecular condensates, possibly due to the presence of an intrinsically disordered region in CAMKV’s C-terminal half, containing seven tandem repeats of an octapeptide motif that may be directly phosphorylated by DYRK3, and thus, likely regulating the relocalization of CAMKV into specific membraneless organelles.

CAMKV remains a largely understudied protein, and this lack of knowledge is extensive to its localization and functions. Some authors have suggested membrane and/or vesicle localization, although the data supporting such features warrant further corroboration. Moreover, membrane localization could indeed result from cell type-, species-, or context-specific characteristics. We provide evidence demonstrating that CAMKV is homogeneously distributed in the cytosol of interphasic NB cells. When NB cells enter the cell cycle, CAMKV levels increase and become relocalized to the mitotic spindle. Given the reported localization of DYRK3 to the mitotic spindle poles [8] and our observation of endogenous CAMKV and phospho-Thr246 AKT1S1 in the mitotic spindles of dividing NB cells, we hypothesize a critical role for this novel DYRK3/CAMKV/AKT1S1 module in the assembly, maintenance, or dissolution of the mitotic spindle of proliferating NB cells. Finally, although CAMKV was originally predicted to lack a kinase activity, this feature has not been properly addressed. Given that CAMKV is highly enriched in NB cells and tumors, we speculate that novel small molecule inhibitors to specifically suppress the DYRK3/CAMKV module could constitute an innovative therapeutic strategy to fight high-risk neuroblastoma in a very precise and effective manner, a topic that will be the subject of our future efforts.

## Data Availability

Data analysis from public data repositories was from the ‘R2: Genomics Analysis and Visualization Platform’ (https://hgserver1.amc.nl/cgi-bin/r2/main.cgi (accessed between February and May 2022) and from ‘DepMap: The Cancer Dependency Map Project’ (https://depmap.org/portal/ (accessed on 20 January 2023)), as indicated in the text. Data were analyzed using the corresponding webpages’ analysis tools as recommended in the ‘Tutorials’ sections of each platform.

## Supplementary Materials

The following supporting information can be downloaded at: https://www.mdpi.com/article/10.3390/biomedicines12010197/s1, Figure S1. Bonferroni adjusted p-values for the correlations between *DYRK* family members expression and Brain tumors patient survival probability. Figure S2. The same cDNA samples from Figure 2B were subjected to qRT-PCR analysis of *DYRK1A* and *DYRK2* expression (vs. the housekeeping gene *TBP*). Figure S3. The same cells from Figure 2C were processed in parallel for protein extraction and Tetracyclineinducible shRNA system validation by WB. Figure S4. Related to Figure 2D. JF cells were plated for proliferation assays in the absence (DMSO) or presence of 1 μM Harmine. Figure S5. Related to Figure 2. Contralateral healthy kidneys from in vivo Tet-shDYRK3 JF tumor formation experiment showed no significant differences in size/weight. Figure S6. Related to Figure 4A. Human CAMKV protein sequence was analyzed with the online PONDR (Predictor Of Natural Disorered Regions; www.pondr.com) tool, suggesting a highly disordered C-terminal region of ~200 amino-acids (upper left panel). Figure S7. Related to Figure 5C. mCherry-CAMKV NGP cells were left untreated (DMSO; left panels) or treated with the indicated final concentrations of Harmine for 24. Harmine treatments induced a relocalization of CAMKV from a homogeneous distribution into numerous aggregates. Figure S8. Related to Figure 5C. mCherry-CAMKV NGP cells were left untreated (DMSO; left panels) or treated with the indicated final concentrations of the DYRK3 inhibitor GSK-626616 for 24. Figure S9. Related to Figure 5D. JF cells were fixed and processed for fluorescent immuno-staining of endogenous CAMKV by laser confocal microscopy, confirming a clear mitotic spindle localization of CAMKV in cells undergoing cell division. Videos S1–S2; Table S1. shRNA and primer sequences.
