# Supplementary material for: A Novel Druggable Dual-Specificity tYrosine-Regulated Kinase3/Calmodulin Kinase-like Vesicle-Associated Signaling Module with Therapeutic Implications in Neuroblastoma
Source: Biomedicines. 2024 Jan 16;12(1):197. doi: 10.3390/biomedicines12010197 (PMC10813661; doi:10.3390/biomedicines12010197)
Supplement: Supplementary file 1 [file biomedicines-12-00197-s001.zip › DYRK3_CAMKV in neuroblastoma EJR Suppl figs S1-9.pdf]

Supplementary materials

Figure S1

blue=worse	Brain Cohort (number of patients)							score
red=better	Madhavan 550 (REMBRANDT)				TCGA 532 (Glioma)	French 284 (Glioma)	Kawaguchi 50 (Glioma)	
DYRK1A	4.20E-04	4.28E-01	1.90E-02	8.39E-01	7.40E-02		7.07E-01	11
DYRK1B								0
DYRK2	2.20E-04	3.30E-03	3.79E-01			5.70E-02		11
DYRK3	9.00E-13				1.38E-08	8.12E-05	4.04E-03	-44
DYRK4								0

Figure S1
Bonferroni adjusted p-values for the correlations between DYRK family members expression and Brain tumors patient survival probability. Values highlighted in blue denote significant negative correlations (higher expression = reduced survival probability ‘worse’), while values highlighted in red denote positive correlations (higher expression = increased survival probability ‘better’). Blue/red fonts denote non-significant values (bonf. p-val > 5.00E-02). Scores are arbitrary units based on the number of datasets showing one or more significant correlations for a given gene, and the value of such significance. We arbitrarily define that a gene must be significantly correlated to either a better or a worse outcome in at least 2 independent cohorts, and obtain a minimum of 20 points (either positive or negative, respectively), to be considered a significant hit.

Figure S2

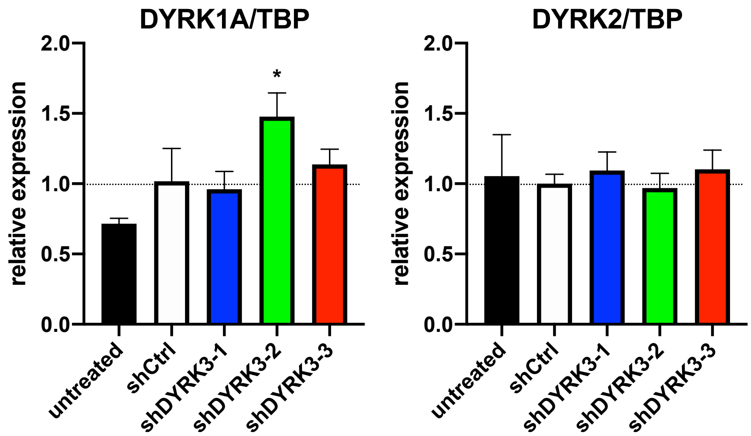

Figure S2
The same cDNA samples from Figure 1B were subjected to qRT-PCR analysis of DYRK1A and DYRK2 expression (vs. the housekeeping gene TBP). No dramatic changes were observed in the expression of other family members upon downregulation of DYRK3.

Figure S3

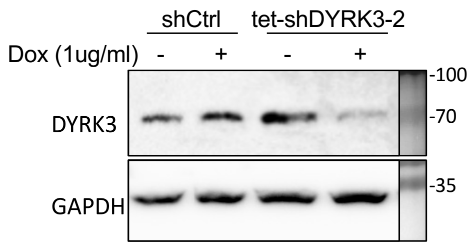

Figure S3
The same cells from Figure 1C were processed in parallel for protein extraction and Tetracycline-inducible shRNA system validation by WB. DYRK3 protein expression was efficiently down-regulated upon doxycycline treatment of Tet-shDYRK3-expressing cells.

Figure S4

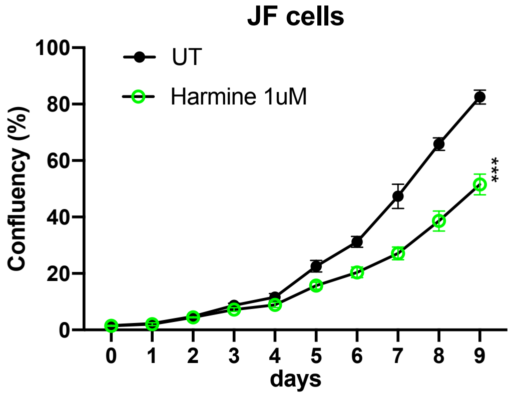

Figure S4
Related to Figure 1D. JF cells were plated for proliferation assays in the absence (DMSO) or presence of 1 μM Harmine.

Figure S5

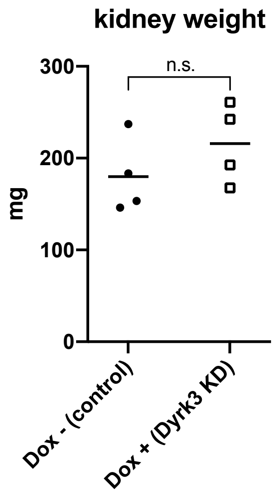

Figure S5
Related to Figure 2. Contralateral healthy kidneys from *in vivo* Tet-shDYRK3 JF tumor formation experiment showed no significant differences in size/weight.

Figure S6

PONDR: Predictor Of Natural Disordered Regions

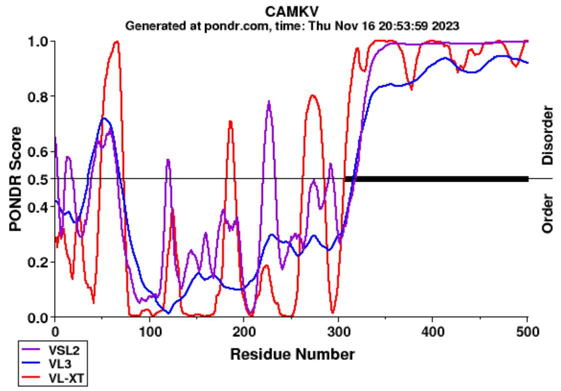

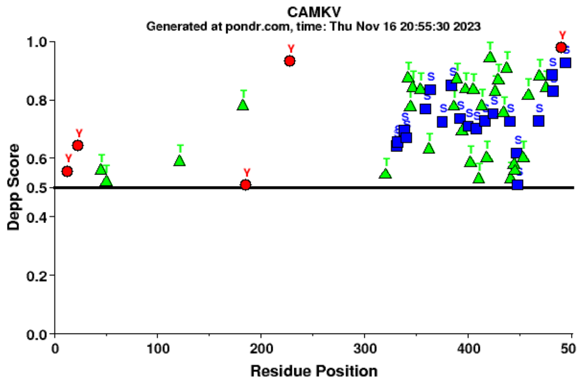

PrDos: Protein DisOrder prediction System

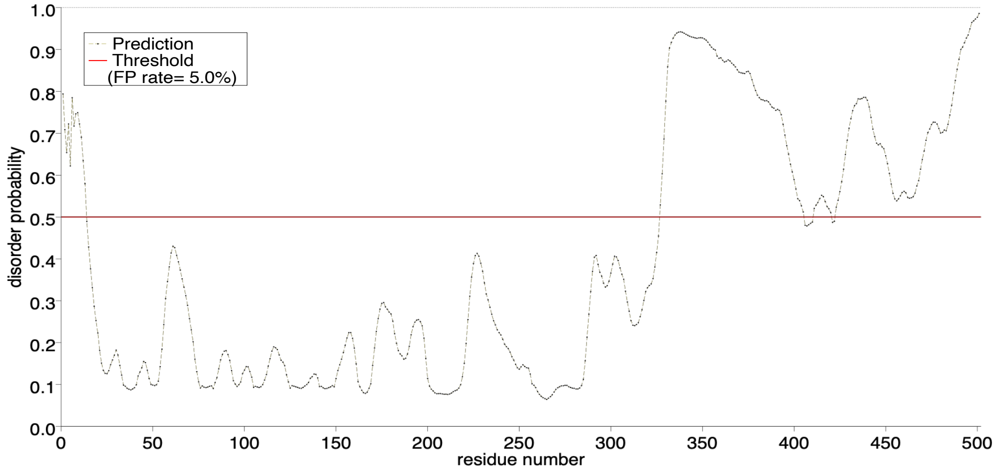

Figure S6

Related to Figure 4A. Human CAMKV protein sequence was analyzed with the online PONDR (Predictor Of Natural Disorered Regions; www.pondr.com) tool, suggesting a highly disordered C-terminal region of ~200 amino-acids (upper left panel). We also used the Disordered Enhanced Phosphorylation Predictor (DEPP) tool from the PONDR webpage. DEPP uses disorder information to improve the discrimination between phosphorylated and non-phosphorylated sites.

Figure S7

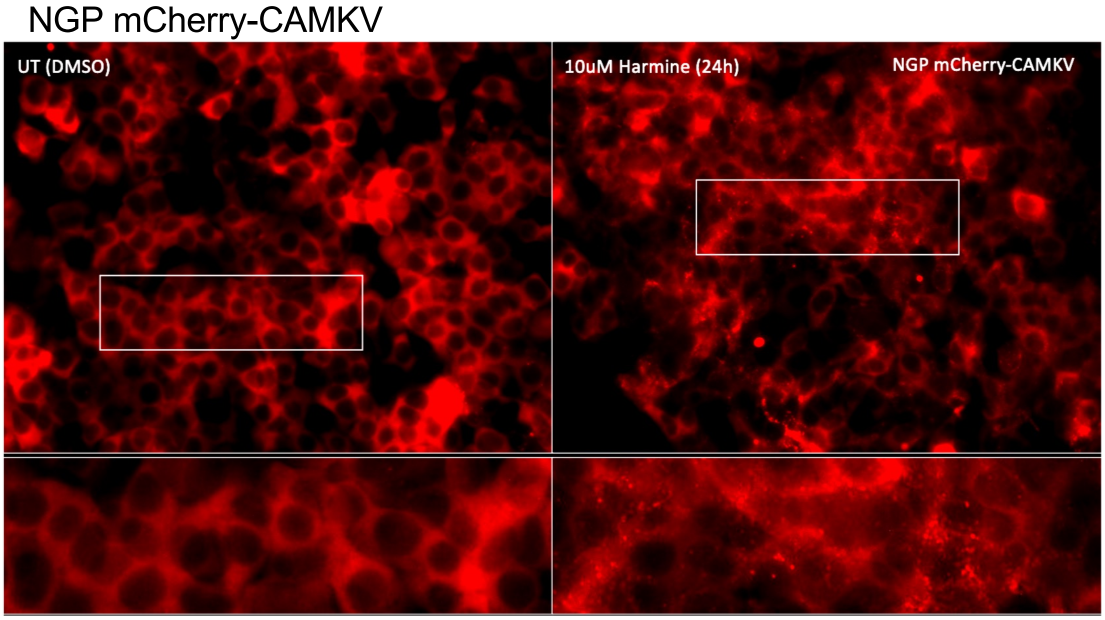

Figure S7
Related to Figure 5C. mCherry-CAMKV NGP cells were left untreated (DMSO; left panels) or treated with the indicated final concentrations of Harmine for 24. Harmine treatments induced a relocalization of CAMKV from a homogeneous distribution into numerous aggregates.

Figure S8

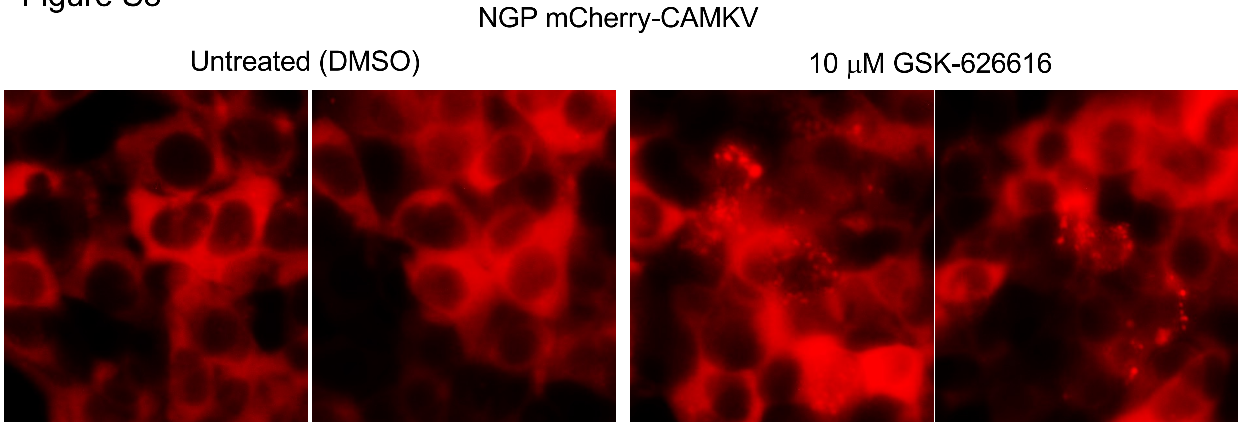

Figure S8
Related to Figure 5C. mCherry-CAMKV NGP cells were left untreated (DMSO; left panels) or treated with the indicated final concentrations of the DYRK3 inhibitor GSK-626616 for 24. GSK-626616 treatment induced a relocalization of CAMKV from a homogeneous distribution into aggregates.

Figure S9

JF anti-CAMKV IF (endogenous) - confocal

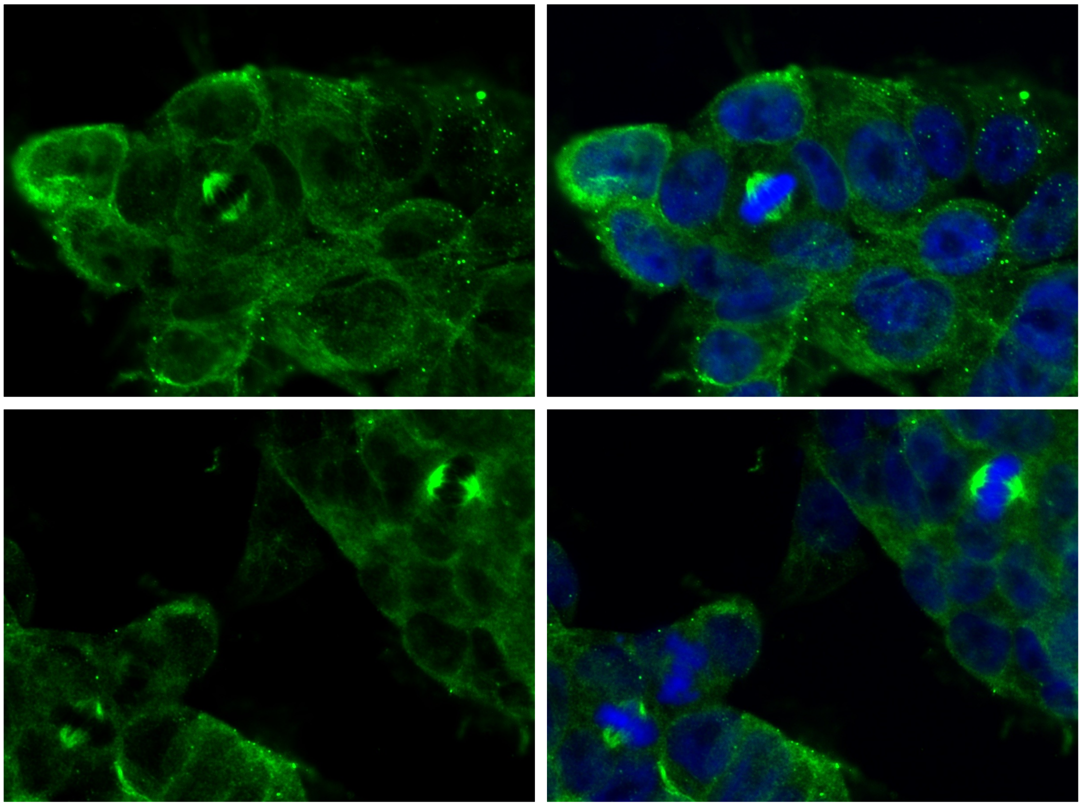

Figure S9

Related to Figure 5D. JF cells were fixed and processed for fluorescent immuno-staining of endogenous CAMKV by laser confocal microscopy, confirming a clear mitotic spindle localization of CAMKV in cells undergoing cell division.
