# Supplementary material for: A Novel Druggable Dual-Specificity tYrosine-Regulated Kinase3/Calmodulin Kinase-like Vesicle-Associated Signaling Module with Therapeutic Implications in Neuroblastoma
Source: Biomedicines. 2024 Jan 16;12(1):197. doi: 10.3390/biomedicines12010197 (PMC10813661; doi:10.3390/biomedicines12010197)
Supplement: Supplementary file 1 [file biomedicines-12-00197-s001.zip › DYRK3_CAMKV in neuroblastoma EJR Suppl table S1.pdf]

shRNA	TRCN code	sequence
shDYRK3-1	TRCN0000000646	ATGATCTTACAAACCTGCAAA
shDYRK3-2	TRCN0000000647	GCCAGGGTCTATGATCACAAA
shDYRK3-3	TRCN0000000649	GTAGGTCCAAATGCCAAGAAA
shCAMKV-1	TRCN0000010254	GCGGAAAGCTGCCAAGAACGA
shCAMKV-2	TRCN0000010255	CGGCTGAAGAACTCGAAGATT
shCAMKV-3	TRCN0000010256	TTGGGATGATATTTCGCAGGC
shCAMKV-4	TRCN0000010257	GAGCAAGACCAGCGGATCACT

Primer name	Notes	sequence
DYRK3 F	Wippich et al., 2013	TCC TTC TGA ACC ACC TCC AC
DYRK3 R		CCT TCA TCT CAC CTC CAT CC
CAMKv Nterm F		TCA CTT CCT CCC TGT GAC CA
CAMKv Nterm R		TTC TTG TCG CCC AGA GTC AC
CAMKv Cterm F		TGG GAT GAT ATT TCG CAG GCA
CAMKv Cterm R		CTC TTC TGC AGT GAT CCG CT
GAPDH F		GTT CGA CAG TCA GCC GCA TC
GAPDH R		TCC GTT GAC TCC GAC CTT CA
TBP F		GCA AGG GTT TCT GGT TTG CC
TBP R		CAA GCC CTG AGC GTA AGG TG
